# Environmental drivers alter PUFA content in littoral macroinvertebrate assemblages via changes in richness and abundance

**DOI:** 10.1007/s00027-023-00996-2

**Published:** 2023-08-31

**Authors:** Ursula Strandberg, George Arhonditsis, Petri Kesti, Jussi Vesterinen, Jussi S. Vesamäki, Sami J. Taipale, Paula Kankaala

**Affiliations:** 1 Department of Environmental and Biological Sciences, University of Eastern Finland, Joensuu, Finland; 2 Department of Physical and Environmental Sciences, University of Toronto, Toronto, Canada; 3 The Association for Water and Environment of Western Uusimaa, Lohja, Finland; 4 Department of Biological and Environmental Sciences, University of Jyväskylä, Jyväskylä, Finland

**Keywords:** Boreal lakes, Littoral benthos, Browning, Eutrophication, Polyunsaturated fatty acids

## Abstract

Shallow littoral areas in lakes are productive and highly diverse ecotonal zones, providing habitats for both invertebrate and vertebrate species. We developed a Bayesian modeling framework to elucidate the relationships between environmental drivers (lake typology, habitat, water chemistry, and latitude) and taxon richness, abundance, as well as the content of polyunsaturated fatty acids (PUFAs) in littoral macroinvertebrate communities in 95 boreal lakes. PUFAs, particularly arachidonic acid (ARA), eicosapentaenoic acid (EPA), and docosahexaenoic acid (DHA), are critical micronutrients to maintain normal physiological functions in consumers. Lake typology was a significant predictor for PUFA content in the invertebrate assemblages, which was connected to taxon richness and/or abundance. Benthic communities in large humus-poor or nutrient-rich lakes displayed higher abundance, taxon richness, and more PUFA-rich taxa, whereas those in medium- and large-sized humic (color 30–90 mg Pt/L) and humus-rich lakes (color >90 mg Pt/L) were characterized by decreased abundance and subsequently low PUFA content. The abundance, taxon richness, and nutritional quality of the communities were also strongly related to latitude. Lakes with lower pH were characterized by lower benthic invertebrate diversity and low frequency of taxa with high somatic EPA and DHA content. The complexity of littoral habitats dominated by various macrophyte assemblages creates an environment that favors higher benthic abundance and increased presence of taxonomic groups with high PUFA content. Nutritional quality of benthic invertebrates for upper trophic levels can be modulated by a complex interplay between external stressors and abiotic factors that typically shape the structure of littoral benthic communities.

**Supplementary Information:**

The online version contains supplementary material available at 10.1007/s00027-023-00996-2.

## Introduction

Shallow littoral areas in lakes are productive and highly diverse subsystems providing temporary or continuous habitats for numerous invertebrate and vertebrate species (e.g., Wetzel 1990; Jónasson 2004; Vadeboncoeur et al. 2011). In comparison with pelagic and profundal invertebrate communities, littoral benthic invertebrate assemblages are generally more diverse (Wetzel 2001; Jónasson 2004). Therefore, littoral communities are included in many biomonitoring programs to evaluate the effects of anthropogenic stressors (acidification, eutrophication, browning) in the lakes. For example, the implementation of the European Union (EU) Water Framework Directive (WFD) requires that littoral macroinvertebrate communities be regularly monitored (Poikane et al. 2016; Järvinen et al. 2019). In addition to taxon richness, biodiversity based on functional feeding and trait groups, as well as phylogenetic distances have also been used for macroinvertebrate community characterization (Johnson and Goedkoop 2002; Heino 2008; Heino and Tolonen 2017) and assessment of human impacts on benthic communities (Twardochleb and Olden 2016).

Although recent studies have significantly advanced our understanding of how natural environmental conditions and human-induced stressors affect littoral macroinvertebrate community structure and biodiversity (e.g., Heino 2008; Lento et al. 2012; McFarland et al. 2010; Tolonen et al. 2017; Johnson et al. 2018), the factors affecting resource availability and nutritional quality of littoral communities for whole-lake food webs have received less attention (but see Lau et al. 2014; Makhutova et al. 2016; Vesterinen et al. 2021). The nutritional quality of the available dietary sources, including polyunsaturated fatty acids (PUFA), impact somatic growth, reproduction, and survival of invertebrates, as well as the trophic transfer of these essential micronutrients to upper trophic levels (Guo et al. 2016; Kühmayer et al. 2020; Strandberg et al. 2020a). Littoral food webs are primarily based on attached algal assemblages, but may also rely on detritus from phytoplankton, macrophytes and terrestrial leaves, as well as on microbial communities responsible for organic matter decomposition (France 1996; Vadeboncoeur et al. 2008; Lau et al. 2014; Crenier et al. 2017; Vesterinen et al. 2022).

In aquatic ecosystems, PUFA from the n-3 and n-6 family are predominantly produced by phytoplankton and periphytic algae. Although the environment may significantly affect the abundance and availability of n-3 and n-6 PUFA in the food web, the most important factors are (i) the phylogenetic origin of the individual taxon, and (ii) the overall biomass of primary producers (Galloway and Winder 2015; Strandberg et al. 2020b); For instance, cyanobacteria and green algae do not contain eicosapentaenoic acid (EPA) or docosahexaenoic acid (DHA), whereas most diatoms are particularly rich in EPA and also contain DHA (Taipale et al. 2013; Strandberg et al. 2015). Another interesting fatty acid is arachidonic acid (ARA), which is less studied than EPA and DHA but critically important in the invertebrate signal transduction system, reproduction, and immune defense (Stanley 2000; Schlotz et al. 2012). The PUFA content in individual macroinvertebrates is determined by the dietary availability and possible metabolic modification (Strandberg et al. 2020a; 2021). Within-taxa evaluations of consumer PUFA typically reveal significant differences due to variations in the diet or the role of environmental drivers; For instance, EPA content in common boreal macroinvertebrate taxa, *Asellus aquaticus* (Arthropoda; Crustacea) as well as chironomids (Arthropoda; Insecta; Chironomidae), and oligochaetes (Annelida; Oligochaeta), appears to be negatively correlated with dissolved organic carbon (DOC) concentrations (Kesti et al. 2022; Vesterinen et al. 2022). However, these within taxa differences are distinctly lower than the differences among different taxa, or even at the order level (Lau et al. 2014; Hiltunen et al. 2015; Vesterinen et al. 2021). From an ecosystem perspective, the changes in biomass and structure of biotic communities, as a response to lake browning or eutrophication, are highly important in determining the overall PUFA abundance and availability in aquatic food webs. Knowledge on the total PUFA availability in littoral communities has practical relevance to lake water quality and fisheries management alike, because littoral ecotones are important nurseries and foraging sites for many fish species, especially for the juvenile stages (Eloranta et al. 2010).

In this study, we aim to investigate the relationships between various environmental drivers (lake typology, habitat, water chemistry, and latitude) with the total abundance of nutritionally important ARA, EPA, and DHA in littoral macroinvertebrate communities, taking into consideration the taxon-specific mass fractions of the above-mentioned PUFA, as well as the taxon richness. The direct environmental effects on PUFA content within a taxon, e.g., due to differences in the diet, are not consider here. Instead, we used taxon-specific mean values and associated variation. We applied a Bayesian modeling framework to characterize the sources of variation in the taxon richness and overall abundance of PUFA, namely EPA, DHA, and ARA, in the littoral benthic macroinvertebrate communities. We hypothesize that the aforementioned environmental drivers influence the taxon richness of benthic macroinvertebrates and subsequently the overall content of PUFA in benthic macroinvertebrate communities.

## Materials and methods

### Biochemical analyses

Fatty acids (FA) were analyzed from benthic macroinvertebrates collected from 25 lakes in Southern and Eastern Finland, with a total surface area varying from 0.009 to 248 km^2^ (Table 1). Total phosphorus (TP) and nitrogen (TN) concentrations ranged between 3–80 and 220–1,250 µg/L, respectively. Lake water color varied from 5 to 447 Pt mg/L, which corresponds to DOC concentration variation of ca. 4–43 mg/L (c.f. Kortelainen 1993). The samples were collected between July and September in 2013 and 2018. The data from the 2013 sampling campaign and those from the 2018 campaign for pond slater (*Asellus aquaticus*), chironomids, and oligochaetes have been previously published (Kesti et al. 2022; Vesterinen et al. 2022). Taxon-specific average values were used for all replicate samples, including samples from lakes studied in both 2013 and 2018.

**Table 1:** Location, surface area, color, and nutrient concentrations (total nitrogen and total phosphorus) of lakes sampled for the fatty acid analyses

	Coordinates EUREF-FIN/WGS84		Area	Color	Tot-N	Tot-P
Lake	Latitude	Longitude	(ha)	(mg Pt/L)	(µg/L)	(µg/L)
Kuorinka	62.61964	29.40788	1292	5	220	3
Syrjänalunen	61.19369	25.14210	0.9	8	352	3
Kajaanselkä	61.13875	25.45200	4271	10	318	19
Karjalan Pyhäjärvi	61.89720	29.95707	24800	16	252	6
Enonselkä	61.02783	25.58533	6470	18	480	31
Valkea Mustajärvi	61.21866	25.11788	13	20	300	10
Pesosjärvi	66.29674	29.50836	44	29	250	5
Kermajärvi	62.44644	28.67387	8551	29	380	5
Ylinen	62.59568	30.22447	369	35	360	3
Kuontijärvi	66.15258	29.00678	595	38	464	19
Tuusulanjärvi	60.43653	25.05332	600	44	1090	79
Kuhajärvi	65.92185	26.68548	305	64	733	35
Pääjärvi	61.05781	25.12747	1344	71	1250	9
Viipperonjärvi	62.50525	27.01991	98	83	393	13
Koitere	62.97302	30.74895	16367	91	316	10
Ätäskö	62.04332	29.98910	1389	103	725	24
Valkea-Kotinen	61.24214	25.06347	4	123	443	18
Hattujärvi	62.95673	31.19679	515	146	402	22
Haapajärvi	63.59774	26.93905	2588	155	890	80
Harkkojärvi	62.95522	31.03665	437	175	415	17
Viitaanjärvi	63.58286	27.31659	361	185	638	47
Nuorajärvi	62.67948	31.12714	4024	193	408	18
Mekrijärvi	62.76192	30.95938	822	203	677	24
Majajärvi	61.12917	25.08359	3.8	414	736	24
Horkkajärvi	61.12860	25.09518	1.1	447	852	38

Littoral benthic macroinvertebrates were collected with the standard kick-netting method from three separate locations, representing main shore types of the lake basin. Macroinvertebrates were separated to different taxa immediately after sampling and stored at −20 °C (Table 2). Frozen macroinvertebrates were lyophilized and ground with a mortar and pestle; 1–6 mg of lyophilized samples were weighed and analyzed for fatty acids. Lipids were extracted twice with chloroform:methanol (2:1) according to Folch et al. (1957). The extracted lipids were transmethylated using 1% H_2_SO_4_ in methanol as catalyst and heating at 90 °C for 90 min. The fatty acyl methyl esters were extracted twice with *n*-hexane, and the final volume was adjusted to either 500 µl (terrestrial dried leaves) or 1 ml (rest of the samples). The samples were run with Shimadzu Ultra (Kyoto, Japan) with Phenomenex ZB-FAME column (length 30 m diameter 0.25 mm, film thickness 0.2 µm) with 5 m guard column. We used splitless injection, and the method followed was based on an initial temperature 50 °C, which was maintained for 1 min, and then increased by 10 °C/min until reaching 130 °C, followed by 7 °C/min temperature increase to 180 °C and then 2 °C/min to 200 °C that was held constant for 3 min. Finally, the temperature was increased by 10 °C/min to 260 °C. Identification of peaks was based on mass spectra and retention time. We used saturated fatty acid 23:0 (Nu-Chek Prep) as an internal standard and GLC 566c (Nu-Chek Prep) as a calibration standard to calculate fatty acid concentrations. The results are presented as mass fractions (µg FA/mg DW).

**Table 2:** Mass fractions (µg/mg DW) and classes (Cl 1-5) of ARA, EPA, and DHA in littoral invertebrates collected from different types of lakes across Finland. Samples from class Insecta represent larval stages unless otherwise indicated. Sample number (*n*) refers to the number of sampled lakes; several replicates within lakes were analyzed when possible. For class limits based on quartiles, see Supplementary Table S1

	Taxon	*n*	ARA (µg/mg DW)			EPA (µg/mg DW)			DHA (µg/mg DW)		
			Mean	SD	Cl	Mean	SD	Cl	Mean	SD	Cl
Platyhelminthes		5	4.3	1.5	5	9.3	4	5	0.9	0.8	5
Nematoda		1	0.6	–	2	5.7	–	3	0	–	1
*Annelida*											
	Oligochaeta	16	3.4	1	4	7.1	2.2	4	0.4	0.2	5
	Hirudinea	12	4.3	1.3	5	5.1	2.6	2	0.2	0.1	4
*Mollusca*											
	Gastropoda	8	2.5	1.6	2	2.7	2.4	2	0.3	0.2	5
	Bivalvia	2	2	0.1	2	0.6	0	2	1.1	0.2	5
*Insecta*											
	Heteroptera/Corixidae	5	3.3	1.3	4	12.1	4.7	5	0.3	0.3	5
	Odonata/Anisoptera	8	2.6	1.1	2	5.2	5	2	0.1	0.1	2
	Odonata/Zygoptera	16	5.3	2.4	5	13.2	7	5	0.2	0.1	4
	Coleoptera/Dytiscidae	7	2.7	1.2	3	7.7	6.4	4	0.2	0.2	4
	Coleoptera (others)	7	3.9	2.9	5	7.2	3.2	4	0.2	0.2	3
	Ephemeroptera	22	3.2	1.8	4	10.2	7.1	5	0	0.1	1
	Trichoptera	17	2.7	1.6	3	6.5	3.2	3	0.1	0.1	3
	Plecoptera	4	2.9	0.7	3	7.4	2.6	4	0.1	0.1	3
	Diptera/Ceratopogonidae	13	2.2	0.8	2	7	2.7	4	0.1	0.1	3
	Diptera/Chironomidae	25	2.7	1.3	3	6.8	3	3	0.1	0.1	3
	Diptera/Tabanidae	3	1.7	0.7	2	7.1	4.4	4	0	0	1
	Diptera/Sialidae	9	3.1	1.7	4	5.7	3.3	3	0.1	0.1	3
	Diptera (others)	2	3.5	1.5	4	8	2.9	5	0.1	0.1	3
*Arachnida*											
	Acari	21	3.2	1.4	4	6.5	3.3	3	0.3	0.3	5
	Dictyonidae (*Argyroneta aquatica*)	4	3.1	0.7	4	6.9	0.6	3	0.2	0.2	3
*Crustacea*											
	Isopoda (*Asellus aquaticus*)	24	4	1.4	5	5	2.6	2	0.7	0.2	5
	Amphipoda/Gammaridae	5	2	1.9	2	3.7	3.5	2	1	0.8	5
	Amphipoda/Pallasidae	2	4.4	3	5	14.4	10.6	5	4.6	3.9	5

We calculated taxon-specific means and standard deviations for ARA, EPA, and DHA mass fractions across all sampled lakes (Table 2), which were then used to establish proxies of the PUFA relationships with environmental drivers. In addition to the direct influence from environmental variables, fatty acids demonstrate very strong dependence on the taxonomic resolution (genus, family, or class), depending on the number of species included (Vesterinen et al. 2021; Kesti et al. 2022), and thus considerable variability was registered within each of the classification groups reported in our study (Table 2). By analyzing fatty acids from 25 different lakes, we aimed to establish robust estimates of taxon-specific mean values and associated standard deviations. The latter variability stemmed from two main factors: (i) the PUFA differences among the different taxonomic units (e.g., species, genera) included within each group, and (ii) the impact of the prevailing environmental conditions on the PUFA content of any given taxon across the different lakes. Because of the relatively small size, the signature of the direct impact of the two factors cannot be disentangled. Moreover, the number of lakes where each taxon was recorded varied significantly, and therefore the degree of our confidence for the corresponding summary statistics differed. To address these shortcomings, we opted for a categorical scheme whereby each taxon was assigned to different classes 1–5 (Table 2), based on the quartiles of the ARA, EPA, and DHA mass fraction values registered in our dataset, as follows: *class 1* representing not detected values, *class 2* accounting for the first quartile, *class 3* denoting the second quartile, *class 4* the third quartile, and finally *class 5* for the fourth quartile. The ranges for all the classes are presented in Supplementary Table S1. We used these categories to classify the taxon-specific PUFA content in the littoral benthic communities. For example, all Plecoptera species were assigned to class 3 in regard to DHA, class 4 for EPA, and class 3 for ARA, whereas all Trichoptera species were consistently classified to class 3 for the three PUFAs examined. This categorical characterization of the PUFA content of each taxon was subsequently combined with the corresponding abundances obtained from the national database (see next section) to obtain the response variables used for our modeling exercise, which were then used to disentangle the relative contribution of a multitude of environmental factors to the total PUFA variability. The overall PUFA content in the littoral communities is expected to be strongly related to the presence and abundance of taxa assigned to PUFA-rich categories, i.e., classes 4 and 5.

### Community composition and lake water chemistry

Data for the littoral macroinvertebrates were compiled from the open database (Hertta) of the Finnish Environment Institute (https://www.syke.fi/avoindata). The lakes considered were classified based on their size, depth, humic content, calcium, and nutrient concentrations (Supplementary Table S2). The shallow littoral areas were sampled with the kick-net standard method (SFS-EN 28265 or SFS 5077; Järvinen et al. 2019) during the autumn (September–October) and, in most cases, the taxa were identified at genus or species level. We focused on lakes in Southern and Eastern Finland sampled during the 2010–2018 period, including the regions of Uusimaa, Häme, Southeastern Finland (Kaakkois-Suomi), Southern Savonia (Etelä-Savo), Northern Savonia (Pohjois-Savo), Central Finland (Keski-Suomi), Northern Carelia (Pohjois-Karjala), Kainuu, Northeastern Ostrobothnia (Pohjois-Pohjanmaa), and Southeastern Lapland (i.e., the same regions from which the samples for FA analyses were collected). For each WFD lake type, 10–12 lakes were randomly selected, except for calcium-rich lakes and large humic lakes, where all sampled locations (3 and 6, respectively) in the dataset were used (Supplementary Table S3, data retrieved during March–June 2020). Our dataset collectively comprised 95 lakes. The number of sampling sites and years varied among the lakes. Our target was to obtain data from at least three randomly selected sites and/or sampling years (2010–2018) per lake. If the sampling was done at ≤ 3 sites/times, all available data for the corresponding lake were selected. During the study period in most lakes (67%) the ecological status was evaluated as good or high, but in 26% and 7% of the lakes the status was moderate or poor, respectively (https://www.syke.fi/avoindata). The water chemistry predictors used were TN, TP, color, and pH. Lake-specific values are averages for the ice-free period (May–October) at 1 m depth. Data used for large lakes (surface area larger than 50 km^2^) were from the sites close to the littoral benthos sampling areas.

### Data analysis

#### Permutational analysis of variance

We used permutational multivariate analysis of variance (PERMANOVA) to study the differences in the taxon-specific PUFA values. The mass fractions of ARA, EPA, and DHA were log(*x* + 1) transformed prior to the analysis. The effect size, i.e., the proportion of variation that was explained by the taxon, was calculated from the estimates of components of variation. PERMANOVA was done by using Primer 6 with the PERMANOVA+ add-on (Anderson et al. 2008).

#### Bayesian modeling framework

The interplay among benthic macroinvertebrate community structure, PUFA content, and prevailing abiotic conditions was depicted in our modeling framework by combining the categorical characterization of the PUFA content of each taxon with the corresponding abundances in each of the 95 lakes selected from the Finnish national database as follows: (i) the total abundance of the benthic community after weighting the abundance ${S}_{n}$ of each taxonomic group *n* by the corresponding PUFA somatic content (${FA}_{n}$) or ${Abundance}_{FAweighted}=\frac{\sum_{n=1}^{N}{S}_{n}x{FA}_{n}}{\sum_{n=1}^{N}{FA}_{n}}$, where *N* represents the total number of taxonomic groups included in our dataset, (ii) the total PUFA content of the benthic macroinvertebrate community after weighting the somatic content of each taxonomic group by the corresponding abundance or ${FA}_{Abundanceweighted}=\frac{\sum_{n=1}^{N}{S}_{n}x{FA}_{n}}{\sum_{n=1}^{N}{S}_{n}}$, (iii) the sum of the products of the taxonomic group abundance with their PUFA somatic content or $FA x Abundance=\sum_{n=1}^{N}{S}_{n}x{FA}_{n}$; the count of taxonomic groups belonging to (iv) classes 4 and 5 in terms of their PUFA somatic content or $Count>3$; and (v) solely to class 5 or $Count>4$. The latter two proxies were also expressed as percentages in an attempt to characterize the presence of PUFA-rich taxonomic groups relative to the total richness of the benthic macroinvertebrate communities recorded across the littoral zones of Finnish lakes. These response variables were separately calculated for ARA, DHA, and EPA.

Our statistical framework is based on an analysis of covariance (ANCOVA) model aiming to characterize the different sources of variation underlying the aforementioned response variables of interest. We used both categorical (vegetation type, substratum of the littoral zone, and lake classification group based on Finnish typology) and continuous (latitude, color, pH, TP, TN) covariates. We also introduced a term that accounted for samples collected from different sites of the littoral zone of the same lake by treating them as repeated measures nested within the same aggregate (contextual) unit. We opted for Bayesian inference to characterize the relative importance of the different predictors, whereby each parameter is treated as a random variable and the likelihood function is used to express the relative plausibility of different parameter values given the available data in our dataset (Gelman et al. 2013). The mathematical notation for the different adaptations of our modeling framework can be summarized as (Arhonditsis et al. 2019):

$$\mathit{ln}\left({\widehat{C}}_{ij}\right)={\beta }_{0i(j)}+{\beta }_{1k\left(ij\right)}+{\beta }_{2l(ij)}+{\beta }_{3m(ij)}+\sum_{n>3}{\beta }_{n}{ln[X}_{nij}]$$

$${X}_{n}=latitude,color, pH, TP,TN$$

$$\mathit{ln}\left({C}_{ij}\right)\sim N(\mathit{ln}\left({\widehat{C}}_{ij}\right),{\sigma }^{2})$$

$${C}_{ij} \sim Poisson({\widehat{C}}_{ij})$$

$$\mathit{logit}\left({\mu }_{ij}\right)={\beta }_{0i(j)}+{\beta }_{1k\left(ij\right)}+{\beta }_{2l(ij)}+{\beta }_{3m(ij)}+\sum_{n>3}{\beta }_{n}{ln[X}_{nij}]$$

$${C\%}_{ij} \sim Bin({\mu }_{ij},{Richness}_{ij})$$

$$\sum_{k=1}^{K}{\beta }_{1k}=0 \sum_{l=1}^{L}{\beta }_{2l}=0 \sum_{m=1}^{M}{\beta }_{3m}=0$$

$${\beta }_{0i(j)}\sim N\left({\beta }_{0j},{\sigma }_{0j}^{2}\right), {\beta }_{1k}\sim N\left(\mathrm{0,10000}\right), {\beta }_{2l}\sim N\left(\mathrm{0,10000}\right), {\beta }_{3m}\sim N\left(\mathrm{0,10000}\right)$$

$${\beta }_{0j}\sim N\left({\mu }_{0},{\tau }_{0}^{2}\right), {\sigma }_{0j}^{-2}\sim G\left(0.001, 0.001\right)$$

$${\mu }_{0}\sim N\left(\mathrm{0,10000}\right) {\tau }_{0}^{-2}\sim G\left(0.001, 0.001\right)$$

$$\beta_{n} \sim N(0,10000)\,\sigma^{ - 2}\sim G\left(0.001,0.001\right)$$

$${{i }} = { 1} \ldots {{I}}\, \, \,\,\,\,\,\,{{j}}\,\, = { 1} \ldots {{J}},\,\,\,\,\,\,\,{{k }} = { 1} \ldots {{K}}, \,\,\,\, {{l }} = { 1} \ldots {{L}},\,\,\,\,\,{{ m }} = { 1} \ldots {{M}}$$

where ${C}_{ij}$ are the values of the response variable of interest registered at littoral site *i* in lake *j*, which in turn is considered to be a draw from either a normal (${Richness}_{ij}$, ${Abundance}_{FAweightedij}$, ${FA}_{Abundanceweightedij}$, ${FA x Abundance}_{ij}$, ${Count}_{ij}>3$), Poisson (${Count}_{ij}>4$), or binomial distribution (${Count}_{ij}>3$ and ${Count}_{ij}>4$ expressed as percentages, or ${C\%}_{ij}$, relative to the corresponding ${Richness}_{ij}$); ${\beta }_{0i(j)}$ is the intercept specifically assigned to site *i* nested within lake *j*, which in turn is a draw from a normal distribution with lake-specific mean and variance terms denoted as ${\beta }_{0j}, {\sigma }_{0j}^{2}$; ${\mu }_{0}, {\tau }_{0}^{2}$ are the mean and variance values of the hyperparameters for the lake-specific terms ${\beta }_{0j}$; ${\beta }_{1k\left(ij\right)}$, ${\beta }_{2l(ij)}$, ${\beta }_{3m(ij)}$ denote the lake, vegetation, and bottom substratum-specific terms depending on the attributes of each site *i* in lake *j*; ${\beta }_{n}$ represents the regression coefficients for the continuous predictors latitude, color, pH, TP, and TN; and ${\sigma }^{2}$ is the model error variance term. ${\widehat{C}}_{ij}$ denotes the model predictions, while ${\mu }_{ij}$ is the predicted probability for the relative presence of PUFA-rich taxonomic groups. *I*, *J*, *K*, *L*, and *M* are the number of sites *i*, lakes *j*, lake types *k*, vegetation types *l*, and substrate types *m*, respectively; $N\left(\mathrm{0,10000}\right)$ is the normal distribution with mean 0 and variance 10,000, and $G\left(0.001, 0.001\right)$ is the gamma distribution with shape and scale parameters of 0.001. The terms representing the effects of the categorical predictors (vegetation type, substratum of the littoral zone, and lake classification group based on Finnish typology) are constrained to have zero sum to make the models identifiable.

We used the general normal-proposal Metropolis algorithm as implemented in the WinBUGS software (Lunn et al. 2000); this algorithm is based on a symmetric normal proposal distribution, whose standard deviation is adjusted over the first 4000 iterations such that the acceptance rate ranges between 20% and 40%. We used two chain runs of 50,000 iterations, and samples were taken after the Markov chain Monte Carlo (MCMC) simulations converged to the true posterior distribution. Convergence was assessed using the modified Gelman–Rubin convergence statistic (Brooks and Gelman 1998). Generally, we noticed that the sequences converged very rapidly (~5000 iterations), and the summary statistics reported were based on the last 45,000 draws by keeping every 10th iteration (thin = 10) to avoid serial correlation. The accuracy of the posterior parameter values was inspected by assuring that the Monte Carlo error (an estimate of the difference between the mean of the sampled values and the true posterior mean; see Lunn et al. 2000) for all parameters was < 5% of the sample standard deviation.

The determination of the most parsimonious models with respect to the number of covariates used to determine the response variables was based on the deviance information criterion (DIC); a Bayesian measure of model fit and complexity (Spiegelhalter et al. 2002). The DIC is given by

$$DIC=\stackrel{-}{D(\theta )}+{p}_{D}$$

where $\stackrel{-}{D(\theta )}$ is the posterior mean of the deviance, a measure of residual variance in data conditional on the parameter vector *θ*. The deviance is defined as −$2log(likelihood) \,or\, -2log[p(y|\theta )]$; ${p}_{D}$ is a measure of the “effective number of parameters” and corresponds to the trace of the product of Fisher’s information and the posterior covariance. It is specified as the posterior mean deviance of the model $\stackrel{-}{D(\theta )}$ minus the point estimate of the model deviance when using the means of the posterior parameter distributions, i.e., ${p}_{D}=\stackrel{-}{D(\theta )}-D\left(\overline{\theta }\right).$ Thus, this Bayesian model comparison first assesses model fit or model “adequacy” (Spiegelhalter et al. 2002), $\stackrel{-}{D(\theta )}$, and then penalizes complexity, ${p}_{D}$. A smaller DIC value indicates a “better” model.

## Results

### PUFA content of taxa

Using PERMANOVA, we found that taxa could explain 19% of the variation in ARA content, 21% of EPA, and 67% of DHA in benthic macroinvertebrates (Table 3). The residual (within-taxa) variability can be attributed to the coarse taxonomic resolution, mainly at the level of order or family, reflecting the heterogeneity in PUFA content of the multiple species included within each taxonomic group. According to our categorical scheme, only few taxa (Corixidae, Oligochaeta, Pallaseidae, Platyhelminthes, and Zygoptera) were assigned to classes 4 or 5, with high content of all three PUFAs considered, i.e., ARA >3.1 µg/mg DW, EPA >7.1 µg/mg DW, and DHA >0.2 µg/mg DW (Table 2). With the exception of bivalvian molluscs, the EPA content exceeded that of DHA and ARA in the taxa identified across the 25 lakes surveyed. The amphipod Pallaseidae had high mean mass fractions of ARA, EPA, and DHA, and especially the DHA content was 5–50 times higher than in the rest of the littoral macroinvertebrates (Table 2). Because we used quartiles to assign the class categories for each fatty acid, several other taxa also received class 4 or 5 for DHA, even though their DHA mass fractions were much lower than that in Pallaseidae.

**Table 3:** PERMANOVA results for the ARA, EPA, and DHA concentrations in benthic invertebrates. Effect size was calculated from the estimates of variation components

Variable	Source of variation	df	SS	MS	Pseudo-F	P(MC)	Effect size
ARA	Taxa	23	11.755	0.51108	3.3878	0.0001	0.19
	Residual	219	33.038	0.15086			
	Total	242	44.792				
EPA	Taxa	23	27.028	1.1751	3.6578	0.0001	0.21
	Residual	219	70.358	0.32127			
	Total	242	97.387				
DHA	Taxa	23	10.601	0.46092	20.939	0.0001	0.67
	Residual	219	4.8207	0.02201			
	Total	242	15.422				

### Upscaling to lake littoral communities

**Fig. 1:**
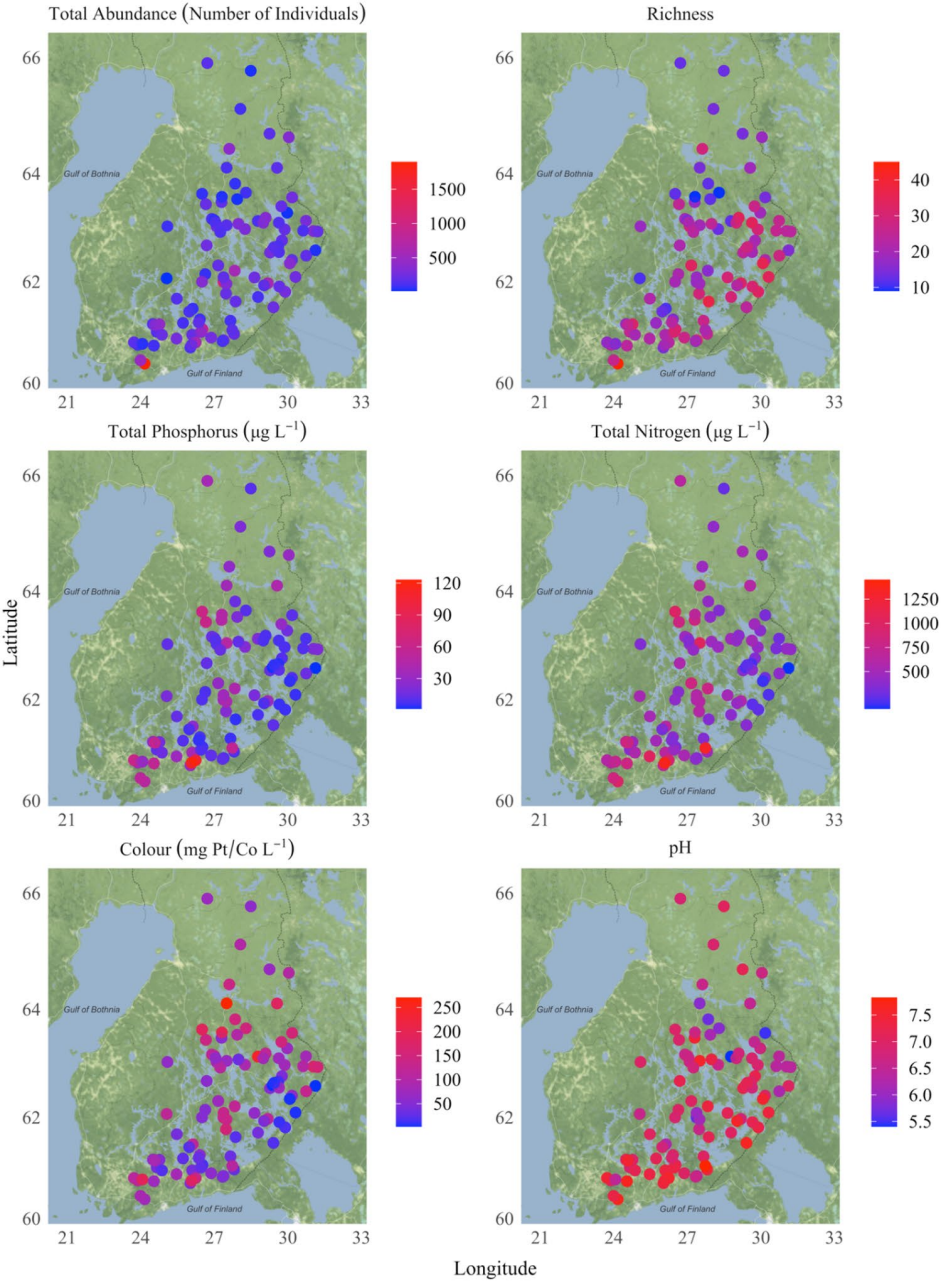
Spatial distribution of abundance and richness of benthic fauna, and water quality across the 95 Finnish lakes considered in our study. Color of the circles indicates (i) the total abundance (number of individuals per sampling effort) of the invertebrate communities, (ii) richness based on the number of taxa registered in each site, (iii) total phosphorus (μg L^−1^), (iv) total nitrogen (μg L^−1^), (v) color (mg Pt/Co L^−1^), and (vi) pH

**Fig. 2:**
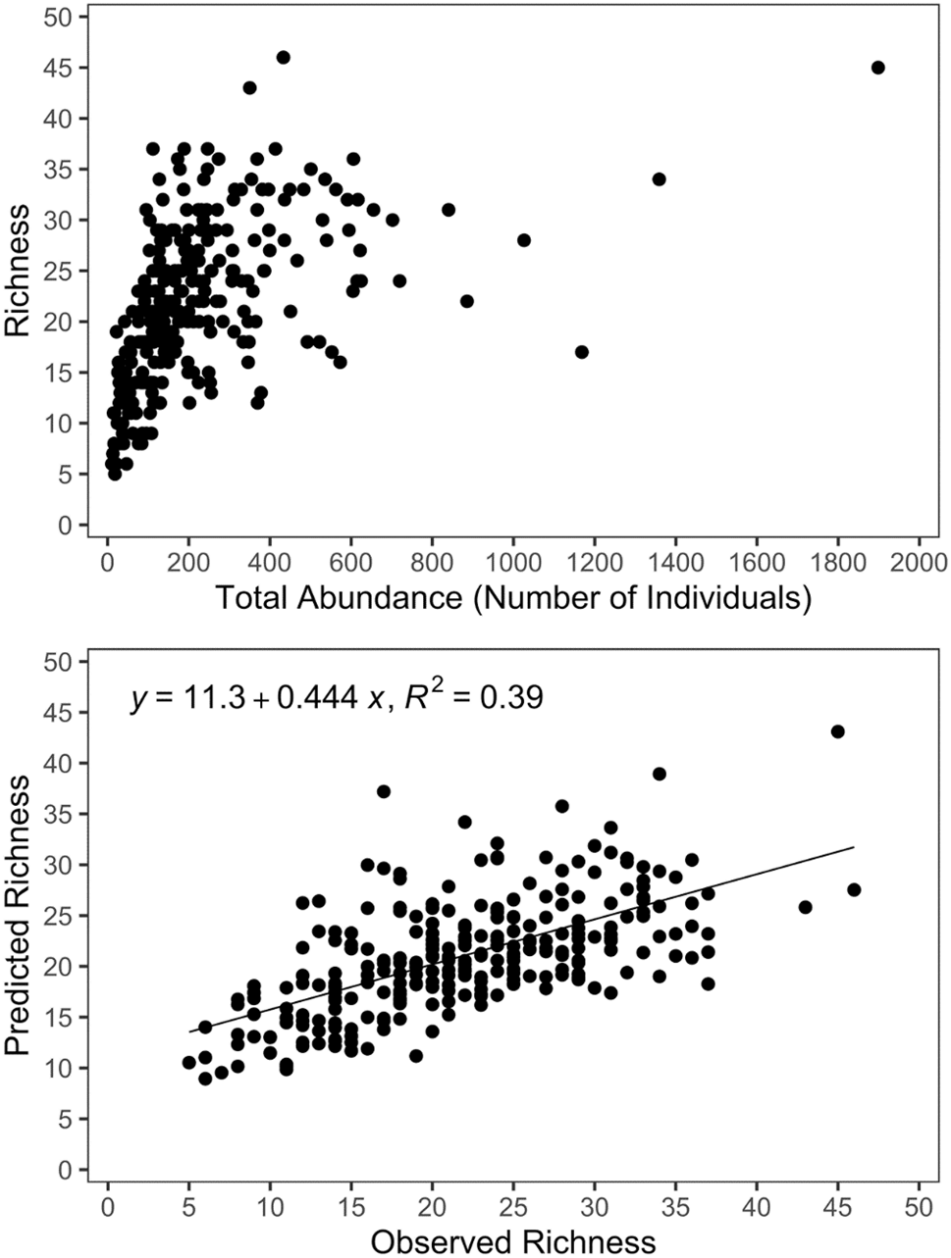
(Top panel) Relationship between richness and total abundance (total number of individuals per sampling effort) of the invertebrate communities across the Finnish lakes studied. (Bottom panel) Performance of the linear log–log regression model, showing that 39% of the richness variability of littoral benthic macroinvertebrate communities can be explained by their corresponding total abundance

**Fig. 3:**
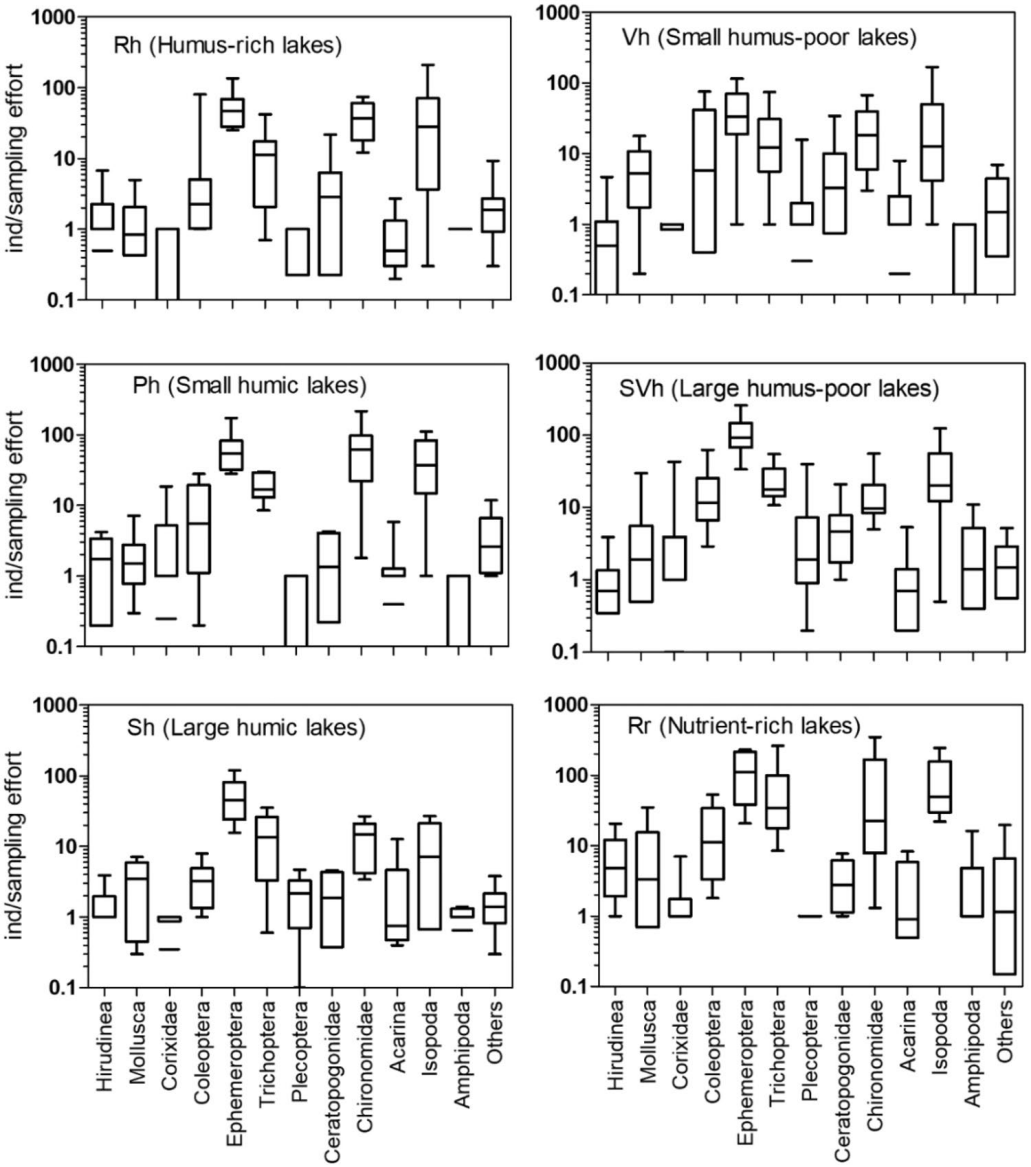
Box plots of the abundance of selected taxonomic groups forming at least 1% of the macroinvertebrate abundance in the monitored lake types, examples for humus-rich, small and large humic lakes, small and large humus-poor and nutrient-rich lakes. Taxa representing <1% were pooled together in the “Others” category

As described in the “Methods” section, the categorical characterization of taxon-specific PUFA levels was used to extrapolate the results from the smaller (*n* = 25) to the larger (*n* = 95) set of lakes selected from the open database of the Finnish Environment Institute. The latter dataset was used to delineate the potential relationships between various environmental drivers (lake type, habitat, water chemistry, and latitude) with the taxon richness, total abundance, and the taxon-specific levels of nutritionally important PUFA in the littoral macroinvertebrate assemblages. Notably, the 95 lakes selected for our modeling analysis displayed significant variability with respect to the total benthic macroinvertebrate abundance (11–1899 individuals per sampling effort) and richness (5–46 taxa), as well as the associated covariates of interest, namely TP (2–123 µg/L) and TN (115–1450 µg/L) concentrations, as well as water color (3 to 271 Pt mg/L) and pH (5.4–7.8) (Fig. 1). Our analysis also showed that the richness of the littoral macroinvertebrate communities displayed a moderately strong relationship with total abundance across the Finnish lakes studied (Fig. 2a). Specifically, the linear log–log regression between the two variables accounted for nearly 40% of the richness variability of littoral benthic macroinvertebrate communities (Fig. 2b). In contrast, the relationship between richness and lake surface area, a surrogate of the ecosystem size, was very weak (*r*^2^ < 5%). Among-lake variation in the benthic macroinvertebrate abundance was prevalent, even for the lakes classified to the same group according to the WFD typology (Fig. 3).

**Fig. 4:**
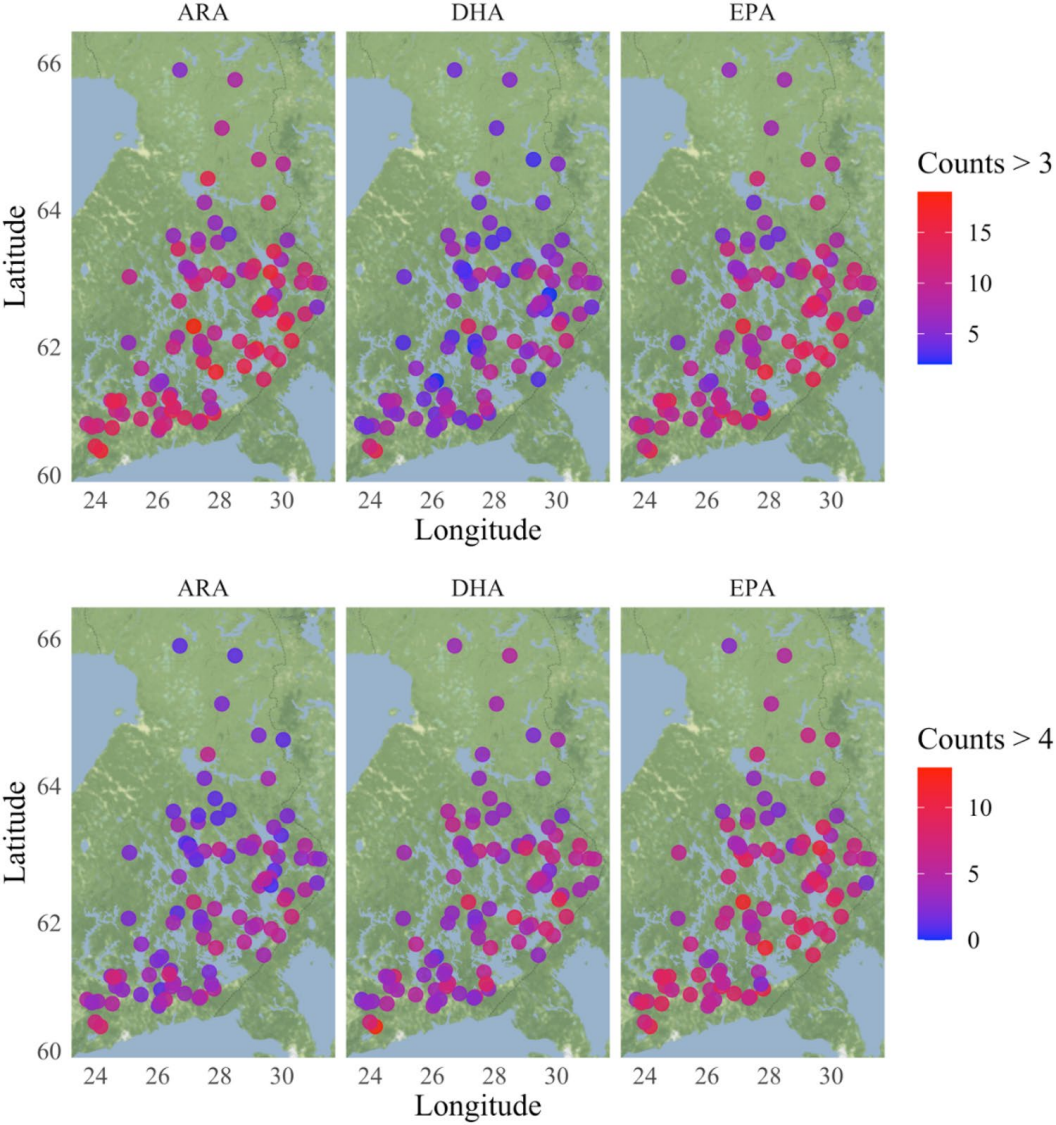
Spatial distribution of PUFA content in benthic fauna across the 95 Finnish lakes considered in our study. Color of the circles indicates the counts of taxa assigned to the different classes in terms of their somatic content in arachidonic acid (ARA), docosahexaenoic (DHA), and eicosapentaenoic (EPA) acids. The upper panels indicate the counts of taxa from classes > 3 (i.e., classes 4 and 5), while the lower panels indicate the counts of taxa from class > 4, (i.e., class 5). Class 4 corresponds to ARA, DHA, and EPA ranges of 3.1–3.6, 0.2–0.3, and 7.0–7.7 μg/mg DW, respectively. Class 5 represents somatic content for ARA, DHA, and EPA greater than 3.6, 0.3, and 7.7 μg/mg DW, respectively

In connecting the two datasets, we were able to approximate the ARA, DHA, and EPA abundance in the macroinvertebrate communities across the Finnish lakes studied, with a tendency of PUFA-rich taxa to register with lower frequency at higher latitudes (Fig. 4). Of the PUFA-rich taxonomic groups, Oligochaeta formed 5–20% of the littoral macroinvertebrate community in the monitored 95 lakes. Many insect taxa (Ephemeroptera, Coleoptera, Plecoptera, Ceratopogonidae, and Tabanidae) were rich in EPA and/or in ARA, but not in DHA (Table 2). Extrapolating to the 95 lakes, Ephemeroptera formed 25–49% and Coleoptera 3–27% of the total macroinvertebrate community and, thus, are potentially significant EPA sources for fish. Chironomids were also prevalent (7–34% of the community) in the littoral area of the lakes, providing a moderate (class 3) source of EPA, DHA, and ARA. Trichoptera, also a moderate source of PUFA, formed 3–15% the community in the studied lakes. The isopod *Asellus aquaticus* was present in 95% of the monitored lakes, in which they accounted for 8–27% of the total macroinvertebrates. *A. aquaticus* was rich in ARA and DHA, but they had relatively low EPA content (class 2). The availability of DHA in the littoral benthic community was rather low, except for Amphipods (Pallaseidae and Gammaridae), which were observed only in 22 of the 95 monitored lakes, mainly in nutrient-rich (*Rr*) and large lakes (*Sh*, *SVh*), representing only 0.1–5% of the total macroinvertebrate community of those lakes.

**Fig. 5:**
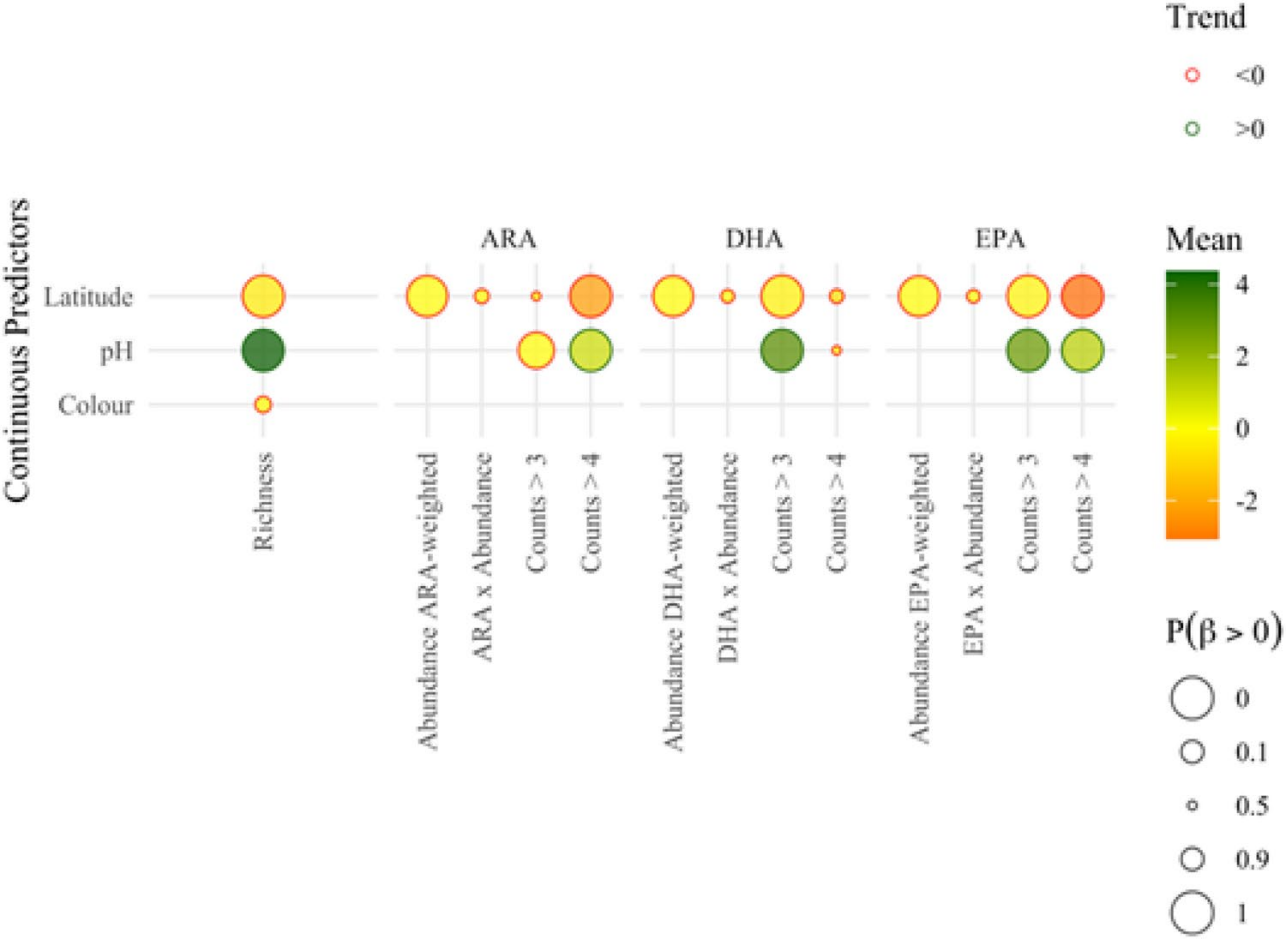
Effects of lake latitude, water color, and pH on richness, ARA, DHA, and EPA profiles of the invertebrate communities across the Finnish lakes studied. A circle is drawn for the posterior regression coefficient between each response and continuous predictor variable, the inner color of which indicates the median of the slope estimate. The size of the circle shows the probability of each slope to be greater than 0, with larger circles nearing to either 0 or 1 and the smaller circles nearing to 0.5. The outline of the circle shows a positive or negative trend as determined by the sign of the posterior slope median. Definitions of all the response variables (labels perpendicular to the *X* axis) are provided in the “Methods” sections

The determination of the most parsimonious models on the basis of their DIC values showed that all the categorical predictors (vegetation type, substrate of the littoral zone, and lake classification group based on Finnish typology) along with the lake latitude were consistently selected in the final model structures. Lake pH was also included in the models intended to describe the frequency of PUFA-rich taxonomic groups in the benthic macroinvertebrate communities, whereas the water color had a discernible relationship only with the richness of littoral benthos (Fig. 5). Considering the presence of PUFA-rich taxonomic groups either as counts (ANCOVA or Poisson models) or as relative frequency (binomial models) led to fairly similar results, although there was a general tendency of the former models to provide parameter posteriors that were better identified relative to the latter ones. Thus, because of their ability to extract more clearly the signature of the examined covariates, our presentation hereafter is based on the modeling of the actual counts of PUFA-rich taxa.

Our modeling analysis revealed a distinctly negative relationship between lake latitude and richness, total benthic macroinvertebrate abundance weighed by the PUFA somatic content of the each taxonomic group (${Abundance}_{FAweighted}$), and presence of PUFA-rich taxa ($Count>4$), which suggests that littoral benthic macroinvertebrate communities in higher-latitude lakes systematically displayed lower richness and abundance, as well as a less frequent presence of taxa classified as having high PUFA somatic content (Fig. 5). Likewise, the positive relationship between pH and richness of littoral benthos or presence of PUFA-rich taxa mainly reflects the lower diversity and frequency of taxa with high PUFA somatic content (especially for EPA) in lakes with lower pH (Fig. 5). Counter to the discernible signature of latitude and pH, water color showed only a weakly negative covariance with the richness of the benthic macroinvertebrate communities and was not included in the optimal (most parsimonious) models for the rest of the response variables examined (Fig. 5). In a similar manner, neither TP or TN lake concentrations were qualified for inclusion in any of our models.

**Fig. 6:**
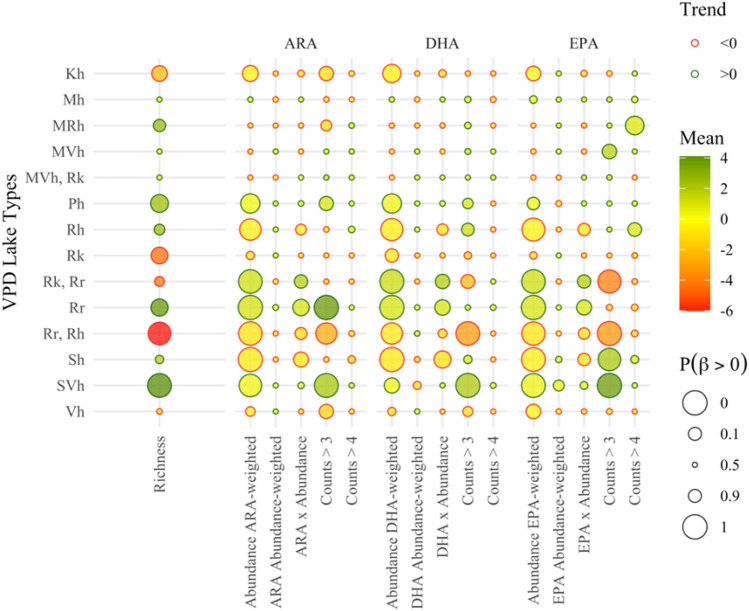
Effects of lake typology on the richness, ARA, DHA, and EPA profiles of the invertebrate communities across the Finnish lakes studied

Medium- (*Kh*) and large-sized humic (*Sh*) lakes were characterized by low PUFA-weighted abundance levels of their benthic macroinvertebrate communities, while the combination of high humic and high nutrient levels (*Rr*, *Rh*) additionally had a distinctly negative impact on their diversity and presence of PUFA-rich taxa (Fig. 6). The latter result specifically stemmed from two lakes (Kirmanjärvi, Niemisjärvi) in the North Savo region dominated by Oligochaeta and Chironomidae. By contrast, small humic lakes (*Ph*) displayed higher richness of their littoral benthic macroinvertebrate communities and a weakly positive relationship with PUFA-weighted abundance levels and presence of PUFA-rich taxa, namely species of the Asellidae (*Asellus aquaticus*) and Caenidae (*Caenis horaria*) families. Large humus-poor lakes (*SVh*) displayed higher richness, PUFA-weighted abundance, and higher prevalence of PUFA-rich benthic taxa, while the same pattern held true for nutrient-rich lakes (*Rr*), especially in terms of their ARA content (Fig. 6). Calcium-rich lakes (*Rk*) displayed lower richness of their littoral benthic macroinvertebrate communities, but no discernible patterns with the corresponding PUFA-weighted abundance and taxa with PUFA-rich somatic content. Interestingly, higher PUFA-weighted benthic macroinvertebrate abundance was registered in the calcium- and nutrient-rich (*Rk*, *Rr*) environment of Lake Valvatus, but with somewhat lower presence of DHA- and EPA-rich benthic taxonomic groups, e.g., species of the Asellidae, Caenidae, and Glossiphoniidae (*Helobdella stagnalis*) families (Fig. 6).

**Fig. 7:**
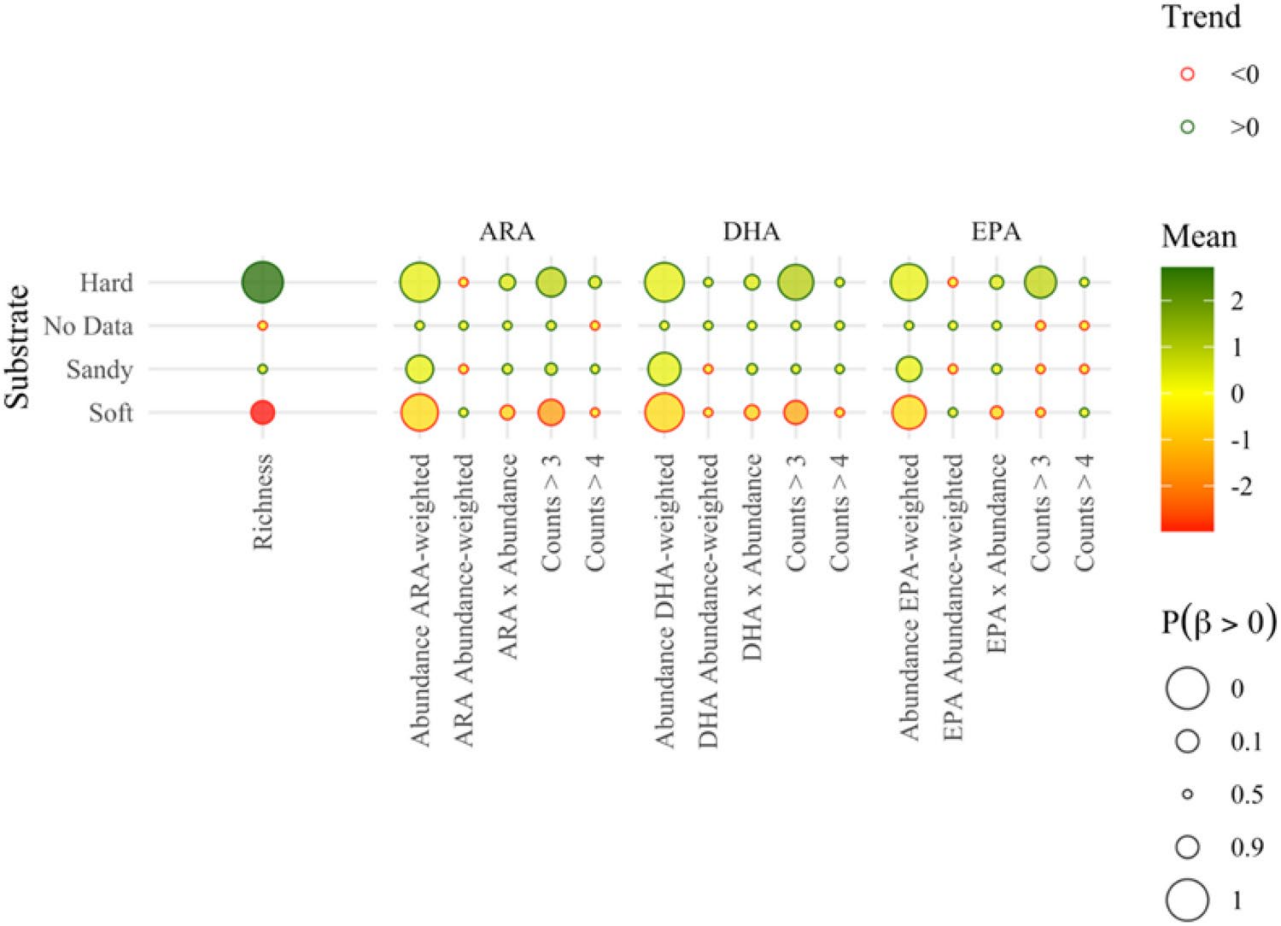
Effects of sediment substrate on the richness, ARA, DHA, and EPA profiles of the invertebrate communities across the Finnish lakes studied

**Fig. 8:**
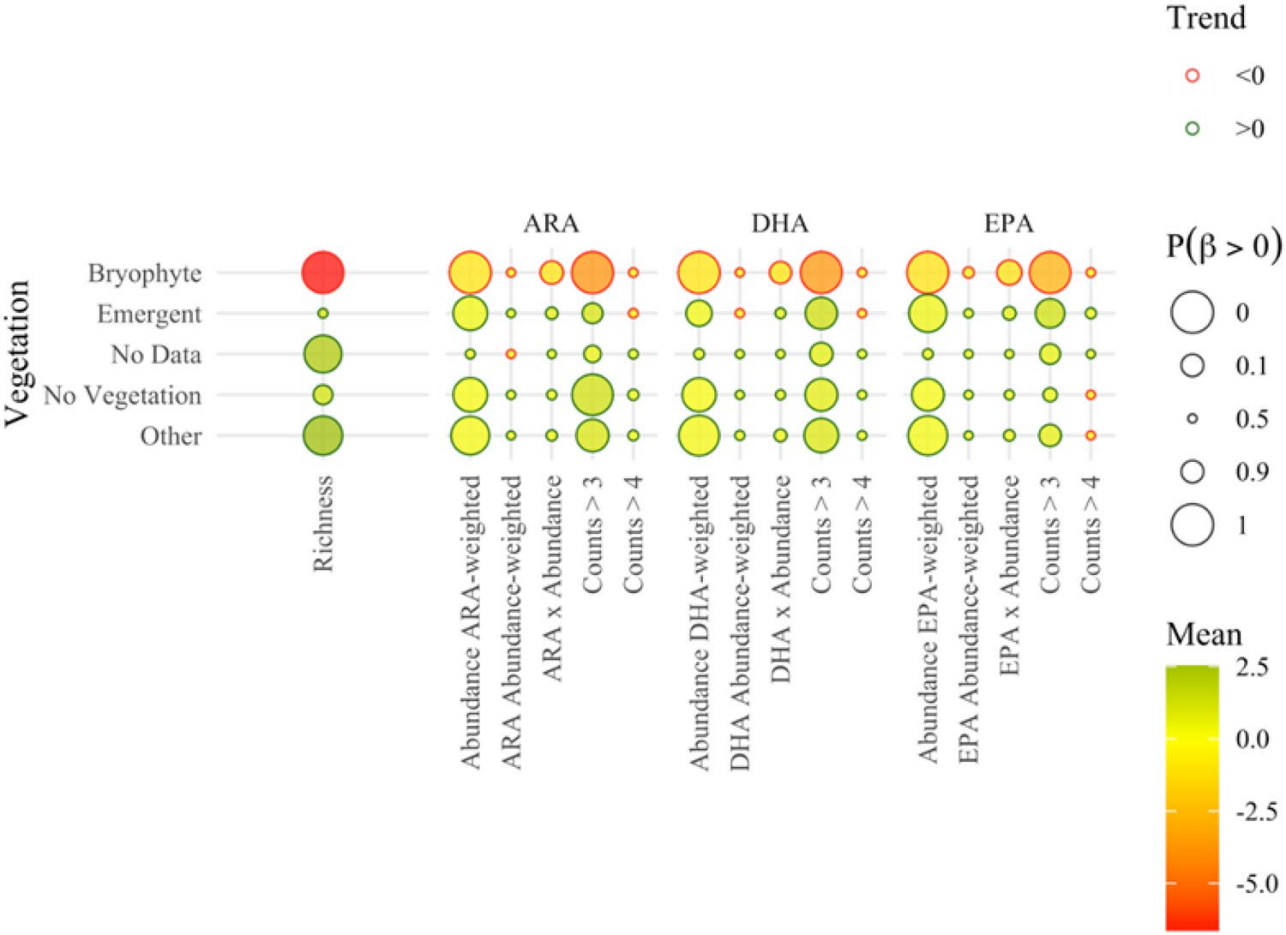
Effects of lake vegetation on the richness, ARA, DHA, and EPA profiles of the invertebrate communities across the Finnish lakes studied

Hard lake-bottom substrata were associated with higher richness and PUFA-weighted abundance of the littoral benthos, as well as with more frequent presence of PUFA-rich taxonomic groups (i.e., see $Count>3$ in Fig. 7). By contrast, soft lake bottom substrata displayed a negative effect on the richness and PUFA-weighted abundance of the benthic macroinvertebrate communities but had a weak (statistically nondiscernible) signature on the prevalence of PUFA-rich taxa (Fig. 7). Our modeling analysis provided evidence of a positive effect primarily on the PUFA-weighted benthic macroinvertebrate abundance and less so on the presence of PUFA-rich taxa in lakes with littoral zones dominated by emergent macrophyte assemblages (Fig. 8). The same pattern held in lakes with littoral habitats characterized by other types of vegetation (e.g., submerged vegetation), which also displayed higher diversity of their benthic macroinvertebrate communities. By contrast, lakes with bryophyte-dominated littoral zone showed a distinctly negative impact on PUFA-weighted abundance, richness, and presence of PUFA-rich taxa.

## Discussion

Owing to their high sensitivity to environmental variations, littoral benthic macroinvertebrate assemblages are an integral component of lake biomonitoring programs to evaluate the degree of the anthropogenic footprint, including the broader impact of acidification (Johnson et al. 2007; McFarland et al. 2010), cultural eutrophication (Donohue et al. 2009; Šidagytė et al. 2013), lake browning (Arzel et al. 2020; Kesti et al. 2022), and landscape features across the shoreline (Brauns et al. 2007; Miler et al. 2013). Consequently, there is a wealth of literature to disentangle the effects of environmental drivers (lake typology, habitat, water chemistry, selective fish predation, and latitude) on the structure and diversity of littoral macroinvertebrate communities (Tolonen et al. 2001; Weatherhead and James 2001; Johnson and Goedkoop 2002; Rennie and Jackson 2005; Heino 2008; Tolonen and Hämäläinen 2010; Lento et al. 2012; Næstad and Brittain 2010; Tolonen et al. 2017; Johnson et al. 2018). However, to the best of our knowledge, this is the first attempt to establish quantitative linkages between the PUFA content of macroinvertebrate communities and environmental drivers. Notwithstanding the uncertainty pertaining to critical assumptions of our analysis, including the coarse taxonomic resolution as well as the extrapolation of benthic PUFA data collected from a subset of 25 lakes to a regional scale (i.e., lakes in Southern and Eastern Finland), our results provide evidence that the lake typology and selected environmental covariates can be significant predictors of PUFA content in the benthic macroinvertebrate communities through their impact on the taxon richness and/or number of individuals (i.e., abundance) of PUFA-rich taxa.

One of the fundamental assumptions of our analysis was that within-taxa variability of PUFA profiles is less pronounced relative to among-taxa differences, and thus the categorical characterization of PUFA composition for individual taxa could be used to draw patterns over broader scales. Phylogenetic position of the benthic macroinvertebrate species along with their feeding strategies are considered to be more important determinants of FA profiles than the prevailing environmental conditions or seasonal variability (Makhutova et al. 2011; Lau et al. 2014; Vesterinen et al. 2021; Hedberg et al. 2023). The taxonomic groups, considered in this study, likely comprised species with different trophic strategies, and as such, the impact of feeding selectivity cannot be unraveled. In terms of the role of phylogeny, larvae of many insect taxa, e.g., Corixidae and Ephemeroptera, are rich in EPA, but typically have much lower DHA content than gammarid crustaceans (Sushchik et al. 2016; Makhutova et al. 2016). Many of these trends are registered across broader geographical regions and appear to be on par with our findings. Our study also found significant among-taxa differences regarding the PUFA profiles (especially for DHA), while the residual variability represents both within-taxa variations and changes on the nutritional content of any given taxon driven by the impact of environmental drivers.

The categorical “lake-type” predictor was consistently included in the most parsimonious models for the abundance, richness, and PUFA content of the littoral benthic macroinvertebrate assemblages. Relative to the numerical average values of nutrient (TN or TP) concentrations or water color, the comprehensive classification of a given lake, on the basis of its size, depth, humic content, calcium, and nutrient concentrations, apparently offers a more sensitive proxy that can characterize the capacity of ecosystem-level conditions to shape the benthic macroinvertebrate communities (Tolonen et al. 2001, Tolonen and Hämäläinen 2010). Because most lakes (67%) in our dataset were evaluated to have a good or high ecological status and the status was poor only in 7%, in comparison with reference conditions in each lake type (https://www.syke.fi/avoindata), we could not directly relate PUFA content in macroinvertebrate communities to human-induced nutrient loading or increased color. This is in accordance with the observation of Alahuhta and Aroviita (2016) for littoral macroinvertebrate communities.

Our analysis showed that nutrient-rich (*Rr*) lakes experience higher abundance (see positive relationships with ${Abundance}_{FAweighted}$ variables) and richness of their benthic macroinvertebrate assemblages. There was no discernible effect on the frequency of PUFA-rich taxa, except from an increase in the presence of taxa rich in their ARA content, such as the pond slater and riffle beetle (*Oulimnius tuberculatus*, Elmidae, Coleoptera). Generally, existing evidence from the literature suggests that nutrient enrichment tends to increase the abundance, alter size, and may induce composition shifts (Blumenshine et al. 1997; Donahue et al. 2009), but the strength of these patterns can be modulated by more granular features of the littoral habitat, including the substratum type and vegetation (Tolonen et al. 2001; Brauns et al. 2007). It is also important to note that, although the high PUFA levels in the macroinvertebrate communities of nutrient-rich lakes may provide much-needed nutrition to fuel fish productivity (Tocher 2010), this benefit may not be sufficient to compensate for the dire ecological ramifications of eutrophication.

The positive relationships between benthic macroinvertebrate abundance and richness disappeared when nutrient-rich lakes also displayed high calcium (*Rk*, *Rr*) or high humic content (*Rr*, *Rh*) levels. The latter lake type has recently received considerable attention owing to the increasing trend of lake browning, associated with increased levels of terrestrial DOC and iron. Reasons for lake browning are diverse and stem from a multitude of factors, such as the catchment soil recovery from acidification, increased precipitation and runoff, shortened soil frost period, and peatland draining (e.g., deWit 2016; Nieminen et al. 2017; Kritzberg 2017; Hayden et al. 2019). Medium- (*Kh*) and large-sized humic (*Sh*) lakes, as well as humus-rich lakes (*Rh*) were characterized by low PUFA-weighted abundance levels of their benthic macroinvertebrate communities. This indicates that increased water color and the associated browning of lakes could decrease the ARA, EPA, and DHA availability to upper trophic level consumers in the littoral food web, as suggested before (Strandberg et al. 2016; Taipale et al. 2016). Lake browning may decrease the abundance of benthic invertebrates (Arzel et al. 2020) by affecting their food sources, e.g., owing to decreased benthic primary production (Vasconcelos et al. 2018), reducing the complexity of the littoral zone as macrophyte density declines (Hilt et al. 2013), and/or by changing their behavior and trophodynamics (Estlander et al. 2009; 2012). Lake browning and the possible decline in benthic invertebrates may be linked to the decline in the number of invertivore waterfowl in recent decades (Pöysä et al. 2019; Elmberg et al. 2020). Interestingly, small humic lakes (*Ph*) in our dataset were characterized by higher richness and higher PUFA-weighted abundance in the macroinvertebrate community. Consistent with the findings reported by Kesti et al. (2022), we found high abundance levels of chironomids (Chironomidae) across most humic lakes, but the smaller ones (*Ph*) also displayed greater abundances for the pond slater, rich in ARA and DHA, and various mayflies (Ephemeroptera), typically rich in ARA and EPA. Mayflies rich in ARA and EPA include *Caenis horaria* (Caenidae), *Leptophlebia marginata* (Leptophlebiidae), and *Kageronia fuscogrisea* (Heptageniidae). Lake browning is a large-scale phenomenon, which may not be captured only by color or nutrient parameters and the impact may be indirect (Creed et al. 2018).

The negative effects of acidic environments on taxon richness and abundance of benthic macroinvertebrates are well documented in both lentic and lotic environments (Økland and Økland 1986; Feldman and Connor 1992; Rosemond et al. 1992; Johnson et al. 1993), across taxa with poor (Anisoptera), moderate (Plecoptera, Trichoptera), and rich (Ephemeroptera) PUFA content. Many boreal lakes in Finland are situated in catchments with large peatland areas and have naturally low pH primarily due to organic acidity (Rantakari and Kortelainen 2008). Our analysis did not include lakes from Western Finland, which are frequently surrounded by watersheds with acid sulfate soils, and these waterbodies may be highly acidic (Nystrand and Österholm 2013). Moreover, littoral environments adjacent to catchments with acid sulfate soils likely experience elevated dissolved metal concentrations, especially in the form of free ions and/or sulfate complexes (Burton and Allan 1986; Nystrand and Österholm 2013). Elevated metal concentrations may be conducive to toxic effects on sensitive taxa (Burton and Allan 1986; Nystrand and Österholm 2013). Nonetheless, while the pH range in our study lakes was somewhat narrower (5.4–7.8), our results signified the likelihood of a negative impact of acidic conditions on taxon richness and frequency of PUFA-rich taxa.

Higher-latitude lakes displayed a discernible pattern of lower abundance, taxon richness, and nutritional quality of their macroinvertebrate assemblages. This finding is on par with existing evidence of a latitudinal gradient in terms of the structural and functional diversity of littoral macroinvertebrate assemblages in Scandinavian lakes (Johnson and Goedkoop 2002; Aroviita et al. 2019). Interestingly, this landscape-level pattern appears to be confounded with the transition zone between the northern coniferous (pine and spruce) and southern mixed forests, as well as a critical geomorphological landscape delineation associated with the highest postglacial coastline, where lakes tend to be nutrient rich, owing to the highest deposition of fluvial sediments (Johnson and Goedkoop 2002). In the Arctic, temperature, together with spatial connectivity, are the driving stressors for the diversity patterns of macroinvertebrate communities (Lento et al. 2022).

The strength of the signal of environmental variations on the composition and integrity of littoral macroinvertebrate assemblages has been shown to vary among habitat types, with the most discernible effects found on stony substrata (Tolonen et al. 2001; Brauns et al. 2007; Donahue et al. 2009). Consistent with the previous studies, our analysis showed that assemblages on hard (stony) substrata distinctly displayed greater richness, abundance, as well as higher frequency of PUFA-rich taxa, while the exact opposite pattern held for soft-bottom habitats in terms of benthic richness and abundance. The same inference about the role of stony substrata could be drawn from the trend in large humus-poor lakes (*SVh*). Large humus-poor lakes were characterized by more abundant and diverse benthic fauna, along with discernibly higher frequency of PUFA-rich taxa, given that the vast majority of the sites classified in this lake type reflected stony habitats. An additional factor that may have reinforced the latter pattern could be the size of the lakes. Large systems are generally assumed to have the propensity for higher taxon diversity due to the widening habitat variability for benthic macroinvertebrate communities (Heino 2008). However, lake size was not a significant predictor for taxon richness, when considering the entire dataset of lakes, with areas ranging from ca. 0.4 to 895 km^2^ and located on a wide geographical area. This reinforces the conclusion that the relationship between lake size and benthic macroinvertebrate assemblage can be significantly modulated by a suite of other biotic components and/or abiotic factors. Even less straightforward is the relationship between the availability of littoral macrophyte beds and the functional diversity of benthic macroinvertebrate assemblages. The composition and complexity of the macrophyte community, as well as the attributes of the surrounding environment, including the water and sediment chemistry around beds, can shape the inter- and intraspecific benthic composition (Twardochleb and Olden 2016). For example, dense macrophyte cover can increase habitat diversity, support taxon richness, and may buffer the effects of wave action in the nearshore environment (Tolonen et al. 2001). In contrast, there is a multitude of processes associated with the prevalence of emergent or floating-leaved macrophytes that can negate the potential benefits of (or even negatively impact) the integrity of the littoral invertebrate communities. These include the reduction of the incident light available to sediments under macrophyte beds, competitive elimination of submerged macrophytes that contribute to the complexity of the littoral habitat owing to severe light limitation, and proliferation of bottom hypoxia due to the decomposition of senescing plant tissues or acceleration of the oxygen respiration rates to the atmosphere (Strayer et al. 2003; Donahue et al. 2009; Twardochleb and Olden 2016; Kim et al. 2021). Although our study did not unequivocally elucidate the details of the relationship between littoral vegetation and benthic fauna, we were able to extract a moderately strong positive signature between both emergent and non-emergent vegetation (“Other” vegetation type in Fig. 8) and benthic macroinvertebrate abundance/presence of PUFA-rich taxa. Surprisingly, we also found a very strong negative correlation between bryophytes and benthic macroinvertebrate richness/abundance, and consequently the overall PUFA content in the littoral macroinvertebrate community. This finding contradicts previous studies reporting that the increased complexity of moss mats could benefit many invertebrate taxa (Henrikson 1993).

One of the possible directions for future improvement of our modeling framework revolves around the use of the “lake-type” predictor as a surrogate variable to represent ecosystem-level conditions. While the distinction of 14 groups allowed for a granular characterization of the lake environment (Fig. 6 and Supplementary Table S2), some lake types were underrepresented in our dataset and, consequently, the potential signature of the corresponding prevailing conditions on the benthic fauna is uncertain. For example, the two types of calcium-rich lakes (e.g., *Rk*, *Rr* or *MVh*, *Rk*) were based on a small number of lakes (Valvatus, Sääksjärvi). Therefore, the somewhat counterintuitively weak impact of elevated calcium concentrations on the richness, abundance, and PUFA content of the macroinvertebrate assemblages may simply stem from the confounding effects of other factors, which we were not able to delineate owing to the small sample size. Preliminary exploratory analysis without considering these lake types did not alter the main conclusions. Nevertheless, the current lake classification system would benefit from accommodating additional ecosystem effects that can potentially improve our predictive capacity (e.g., residence time, ratio of the littoral to total surface area, fetch, lakeshore morphology). In addition, our modeling framework did not explicit consider catchment- (e.g., total area, average slope, land cover, presence of wetlands, hydrological connectivity) or riparian-related (e.g., coniferous, deciduous, arable land, urban) predictors, which can demonstrably shape the littoral invertebrate communities (Johnson and Goedkoop 2002; Alahuhta and Aroviita 2016). Additional classifiers of the habitat complexity (e.g., presence of gobbles, gravels, fine or coarse detritus, detailed characterization of the aquatic vegetation) could have improved the predictive capacity of the model. In view of the ongoing decline in the abundance and diversity of aquatic macroinvertebrate communities, owing to a wide range of external stressors (climate change, browning, acidification, eutrophication), the development of robust modeling frameworks with sound causal foundation has become one of the emerging imperatives for lake and fisheries management alike; our study represents a first attempt to assist in this direction.

## Conclusions

The key finding of this study is that the total PUFA abundance in the benthic macroinvertebrate assemblages is connected to the number of taxa and number of individuals of PUFA-rich taxa in the community. High frequency of PUFA-rich taxa tends to be registered in nutrient-rich lakes, owing to overall higher diversity and greater abundance of benthic macroinvertebrates. Our analysis also showed that the strength of that (presumably) causal relationship can be profoundly modulated by the influence of other environmental stressors, such as lake acidity and browning, as well as geomorphological features of the watershed or the lake itself. Owing to our fundamental assumption to assign group-specific PUFA classes across all lake types, our study was *a priori* not designed to identify specific taxa that may drive the overall PUFA abundance in a lake. Although environmental stressors and seasonal variability have been noted to drive PUFA content within individuals, among-taxa differences in PUFA content have been documented to be discernibly higher than the within-taxa ones. The latter evidence lends support to a fundamental assumption of our analysis, to draw larger scale patterns regarding the total PUFA availability in the benthic macroinvertebrate communities across a diverse range of lakes.

The impact of the structural and functional diversity of the benthic macroinvertebrate community on PUFA availability has broader implications for the integrity of littoral food webs, which are also important foraging grounds for many fish species. Thus, the results of this study provide valuable information for lake and fisheries management, as PUFA availability may become one of the emerging issues in lake and fisheries management, because the number of macroinvertebrates has been decreasing over the past decades.The next compelling question will be to disentangle the tightly intertwined effects of environmental change on both taxa and communities, whereby the environmentally driven variation of PUFA within taxonomic groups can be separated from the variation collectively registered in community assemblages.

### Supplementary Information

Below is the link to the electronic supplementary material:

Supplementary file 1 (DOCX 23 KB)

Supplementary file 2 (DOCX 16 KB)

Supplementary file 3 (DOCX 43 KB)

## Data Availability

Data on fatty acid composition and content of littoral invertebrates are available from the authors upon reasonable request.
