# Supplementary material for: Environmental drivers alter PUFA content in littoral macroinvertebrate assemblages via changes in richness and abundance
Source: Aquat Sci. 2023 Aug 31;85(4):100. doi: 10.1007/s00027-023-00996-2 (PMC10471644; doi:10.1007/s00027-023-00996-2)
Supplement: Supplementary file 1 — (DOCX 23 KB) [file 27_2023_996_MOESM1_ESM.docx]

**SUPPORTING INFORMATION, Suppl. Table S1**

**Environmental drivers alter PUFA content in littoral invertebrate assemblages via changes in richness and abundance**

**Ursula Strandberg^1^, George Arhonditsis^2^, Petri Kesti^1^, Jussi Vesterinen^1,3^, Jussi Vesamäki^1^, Sami J. Taipale^4^, Paula Kankaala^1*^**

**^1^ University of Eastern Finland, Department of Environmental and Biological Sciences, Finland**

**^2^ University of Toronto, Department of Physical and Environmental Sciences, Canada**

**^3^ The Association for Water and Environment of Western Uusimaa, Finland**

**^4^ University of Jyväskylä, Department of Biological and Environmental Sciences, Finland**

* Corresponding author

E-mail: [paula.kankaala@uef.fi](mailto:paula.kankaala@uef.fi), Tel.: 050 431 3496

**Table S1:** Criteria for different PUFA groups of benthic macroinvertebrates (Classes 1-5) based on quartiles (µg/mg DW).

| **Category** | **1** | **2** | **3** | **4** | **5** |
| --- | --- | --- | --- | --- | --- |
| ARA | nd | 0-2.4 | 2.4-3.0 | 3.0-3.6 | >3.6 |
| EPA | nd | 0-5.6 | 5.6-7.0 | 7.0-7.7 | >7.7 |
| DHA | nd | 0-0.1 | 0.1-0.2 | 0.2-0.3 | >0.3 |
| EPA+DHA | nd | 0-5.8 | 5.8-7.1 | 7.1-7.9 | >7.9 |
