# Supplementary material for: Environmental drivers alter PUFA content in littoral macroinvertebrate assemblages via changes in richness and abundance
Source: Aquat Sci. 2023 Aug 31;85(4):100. doi: 10.1007/s00027-023-00996-2 (PMC10471644; doi:10.1007/s00027-023-00996-2)
Supplement: Supplementary file 2 — (DOCX 16 KB) [file 27_2023_996_MOESM2_ESM.docx]

**SUPPORTING INFORMATION, Suppl. Table S2**

**Environmental drivers alter PUFA content in littoral invertebrate assemblages via changes in richness and abundance**

**Ursula Strandberg^1^, George Arhonditsis^2^, Petri Kesti^1^, Jussi Vesterinen^1,3^, Jussi Vesamäki^1^, Sami J. Taipale^4^, Paula Kankaala^1*^**

**^1^ University of Eastern Finland, Department of Environmental and Biological Sciences, Finland**

**^2^ University of Toronto, Department of Physical and Environmental Sciences, Canada**

**^3^ The Association for Water and Environment of Western Uusimaa, Finland**

**^4^ University of Jyväskylä, Department of Biological and Environmental Sciences, Finland**

* Corresponding author

E-mail: [paula.kankaala@uef.fi](mailto:paula.kankaala@uef.fi), Tel.: 050 431 3496

**Table S2:** Lake types based on area, mean depth and water color (mg Pt L^-1^) according to Water Framework Directive (WFD) classification criteria/guidelines in Finland.

| **Lake type** | **Description** | **Area (km^2^)** | **Mean depth (m)** | **Color (mg Pt/L)** |
| --- | --- | --- | --- | --- |
| Vh | Small and medium-sized humus-poor lakes | <40 |  | <30 |
| Ph | Small humic lakes | <5 | >3 | 30-90 |
| Kh | Medium-sized humic lakes | 5-40 | >3 | 30-90 |
| SVh | Large humus-poor lakes | >40 |  | <30 |
| Sh | Large humic lakes | >40 |  | 30-90 |
| Rh | Humus-rich lakes |  | >3 | >90 |
| MVh | Shallow humus-poor lakes |  | <3 | <30 |
| Mh | Shallow humic lakes |  | <3 | 30-90 |
| MRh | Shallow humus-rich lakes |  | <3 | >90 |
| Rk | Calcium-rich lakes |  |  |  |
| Rr | Nutrient-rich lakes |  |  |  |
| Lv* | Lakes with very short water retention |  |  |  |
| PoLa* | Lakes in northern Lapland |  |  |  |

*Not included in the present analysis
