# Supplementary material for: Environmental drivers alter PUFA content in littoral macroinvertebrate assemblages via changes in richness and abundance
Source: Aquat Sci. 2023 Aug 31;85(4):100. doi: 10.1007/s00027-023-00996-2 (PMC10471644; doi:10.1007/s00027-023-00996-2)
Supplement: Supplementary file 3 — (DOCX 43 KB) [file 27_2023_996_MOESM3_ESM.docx]

**SUPPORTING INFORMATION, Table S3**

**Environmental drivers alter PUFA content in littoral invertebrate assemblages via changes in richness and abundance**

**Ursula Strandberg^1^, George Arhonditsis^2^, Petri Kesti^1^, Jussi Vesterinen^1,3^, Jussi Vesamäki^1^, Sami J. Taipale^4^, Paula Kankaala^1*^**

**^1^ University of Eastern Finland, Department of Environmental and Biological Sciences, Finland**

**^2^ University of Toronto, Department of Physical and Environmental Sciences, Canada**

**^3^ The Association for Water and Environment of Western Uusimaa, Finland**

**^4^ University of Jyväskylä, Department of Biological and Environmental Sciences, Finland**

* Corresponding author

E-mail: [paula.kankaala@uef.fi](mailto:paula.kankaala@uef.fi), Tel.: 050 431 3496

**Table S3:** Lake type, surface area, location, total phosphorus (TP) and total nitrogen (TN) concentrations (μg L^-1^), colour (mg Pt L^-1^), and pH of the ninety-five (95) Finnish lakes used in this study (Data from (<https://www.syke.fi/avoindata>). Coordinates EUREF-FIN / WGS84.

| **Lake Name** | **Lake type** | **Area (ha)** | **Latitude** | **Longitude** | **TP**  **(µg/L)** | **TN (µg/L)** | **Colour (mg Pt/L)** | **pH** |
| --- | --- | --- | --- | --- | --- | --- | --- | --- |
| Arrajärvi | Kh | 979 | 60.97 | 26.09 | 38 | 644 | 39 | 7.4 |
| Haatajanjärvi | MRh | 73.9 | 64.10 | 29.55 | 44 | 621 | 183 | 6.5 |
| Hahmajärvi | Ph | 93 | 60.90 | 25.45 | 34 | 1013 | 83 | 7.3 |
| Hanhijärvi | MRh | 438.7 | 61.79 | 27.46 | 23 | 614 | 156 | 6.6 |
| Harkkojärvi | Rh | 437.7 | 62.97 | 31.04 | 20 | 423 | 167 | 6.4 |
| Hattujärvi | MRh | 521 | 62.95 | 31.20 | 21 | 414 | 155 | 6.4 |
| Hauhonselkä | Kh | 2218.8 | 61.18 | 24.52 | 28 | 726 | 65 | 7 |
| Haukijärvi | Ph | 155.1 | 63.04 | 27.08 | 11 | 401 | 88 | 6.6 |
| Haukivesi | Sh | 56714.9 | 62.21 | 28.30 | 19 | 507 | 62 | 7.2 |
| Haukkajärvi | Kh | 1312.4 | 60.90 | 26.88 | 12 | 534 | 58 | 7 |
| Heitjärvi | Kh | 779.4 | 63.05 | 25.06 | 12 | 338 | 63 | 7 |
| Heposelkä | SVh | 5318.9 | 62.23 | 29.47 | 14 | 367 | 51 | 7.2 |
| Hietanen | Rh | 69.4 | 63.14 | 28.78 | 21 | 480 | 240 | 5.4 |
| Hiidenvesi | Rr | 2922.7 | 60.37 | 24.23 | 49 | 983 | 76 | 7.7 |
| Höytiäinen | SVh | 28254.8 | 62.87 | 29.45 | 9 | 359 | 44 | 7.2 |
| Iso Heinäjärvi | Vh | 352.7 | 62.09 | 30.35 | 5 | 195 | 5 | 7.4 |
| Iso Paihmas | Vh | 111.3 | 62.15 | 26.63 | 8 | 363 | 48 | 7 |
| Iso Polvijärvi | Vh | 108.6 | 62.68 | 29.59 | 3 | 160 | 10 | 7 |
| Iso Rautavesi | Kh | 856 | 62.07 | 25.05 | 19 | 530 | 117 | 6.6 |
| Iso Vehkajärvi | Mh | 671.6 | 61.98 | 29.17 | 32 | 585 | 114 | 6.8 |
| Iso-Kaihlanen | Mh | 155.3 | 62.02 | 26.49 | 9 | 394 | 50 | 6.9 |
| Iso-Luotikas | Ph | 137 | 61.47 | 26.13 | 28 | 653 | 120 | 6.2 |
| Iso-Melanen | Rh | 37.1 | 64.44 | 27.61 | 28 | 460 | 140 | 6.6 |
| Iso-Ruhmas | Vh | 425.2 | 61.07 | 26.50 | 6 | 320 | 15 | 7.2 |
| Juuanjärvi | Rh | 215.1 | 63.20 | 29.10 | 8 | 380 | 120 | 6.8 |
| Kajoonjärvi | Kh | 550.8 | 63.11 | 28.97 | 8 | 413 | 78 | 6.8 |
| Kangasjärvi | MRh | 1970.8 | 61.99 | 27.38 | 38 | 555 | 136 | 5.6 |
| Kankaistenjärvi | Vh | 278.7 | 61.01 | 24.66 | 11 | 367 | 37 | 6.9 |
| Kaukjärvi | Rr | 202.5 | 60.82 | 23.74 | 63 | 616 | 110 | 7.5 |
| Keplakko | Rk | 151 | 61.93 | 29.02 | 7 | 430 | 43 | 7.6 |
| Kesijärvi | Ph | 283.8 | 60.75 | 24.52 | 55 | 815 | 73 | 7.7 |
| Kiltuanjärvi | Rh | 1011.4 | 63.78 | 27.87 | 14 | 417 | 150 | 5.7 |
| Kinnasjärvi | Rh | 142.4 | 62.50 | 30.75 | 15 | 390 | 130 | 6.2 |
| Kirmanjärvi | Rr, Rh | 313 | 63.48 | 27.31 | 47 | 810 | 53 | 7.5 |
| Kivijärvi (Luumäki) | SVh | 6492 | 60.95 | 27.68 | 8 | 483 | 28 | 7.4 |
| Kivijärvi (N-basin, Lemi) | Kh | 1079 | 61.06 | 27.76 | 30 | 847 | 77 | 7.3 |
| Koitere | Sh | 16312.2 | 62.95 | 30.63 | 9 | 315 | 103 | 6.4 |
| Kostonjärvi | Sh | 4351.2 | 65.80 | 28.44 | 10 | 253 | 59 | 7.1 |
| Kotajärvi | MRh | 89.8 | 63.45 | 26.65 | 61 | 820 | 140 | 6.9 |
| Kuhajärvi | Mh | 309.1 | 65.92 | 26.69 | 38 | 642 | 72 | 6.9 |
| Kuijärvi | Vh | 321.4 | 61.28 | 26.41 | 7 | 373 | 54 | 7 |
| Kuivajärvi (Suomussalmi) | Ph | 146.6 | 64.64 | 30.05 | 30 | 429 | 103 | 6.7 |
| Kuivajärvi (Tammela) | Mh | 823.3 | 60.78 | 23.86 | 41 | 655 | 117 | 7 |
| Kuolimo | SVh | 7977.1 | 61.34 | 27.42 | 6 | 350 | 31 | 7 |
| Kuorinka | Vh | 1299.7 | 62.62 | 29.41 | 2 | 180 | 3 | 7.1 |
| Kyntinen | Mh | 56.6 | 63.16 | 27.00 | 14 | 500 | 110 | 6.7 |
| Kyynelmyksenjärvi | Ph | 107.1 | 60.86 | 27.39 | 7 | 340 | 50 | 6.4 |
| Laklajärvi | MRh | 71.8 | 63.57 | 30.18 | 11 | 315 | 155 | 5.5 |
| Lehee | Mh | 105.6 | 61.17 | 24.78 | 32 | 714 | 45 | 7.3 |
| Leppävesi | SVh | 6406.9 | 62.27 | 25.93 | 18 | 477 | 49 | 7 |
| Luonteri | SVh | 11586.1 | 61.63 | 27.79 | 4 | 350 | 22 | 7.2 |
| Lyhyenjärvi | Rk | 61 | 63.06 | 27.50 | 48 | 1016 | 67 | 7.6 |
| Mainuanjärvi | MRh | 200.5 | 64.11 | 27.47 | 36 | 653 | 271 | 5.9 |
| Muuruvesi | Kh | 1605 | 62.99 | 27.89 | 23 | 463 | 91 | 6.6 |
| Nevajärvi | Mh | 566.1 | 61.96 | 27.51 | 42 | 620 | 135 | 6.1 |
| Niemisjärvi | Rr, Rh | 418.8 | 63.64 | 26.50 | 64 | 987 | 186 | 7 |
| Pääjärvi | Rh | 123.8 | 60.79 | 24.06 | 30 | 742 | 200 | 6.4 |
| Päijänne (central basins) | SVh | 86416.8 | 61.66 | 25.49 | 12 | 533 | 38 | 7.1 |
| Päijänne (southern basins) | SVh | 7686.9 | 61.17 | 25.58 | 10 | 502 | 26 | 7.1 |
| Pieksänjärvi | Mh | 2105.9 | 62.34 | 27.13 | 29 | 820 | 73 | 7 |
| Pielinen | Sh | 89547.4 | 63.26 | 30.08 | 16 | 366 | 71 | 6.7 |
| Pieni-Panka | MRh | 73 | 63.18 | 26.91 | 14 | 465 | 120 | 6.6 |
| Pihlajavesi | Sh | 72584.5 | 61.75 | 29.38 | 8 | 382 | 38 | 7 |
| Puruvesi | SVh | 41983.3 | 61.96 | 29.70 | 5 | 221 | 10 | 7.2 |
| Pusonjärvi | Ph | 165 | 62.99 | 29.87 | 8 | 419 | 79 | 6.9 |
| Pusulanjärvi | Rr | 211.3 | 60.46 | 23.99 | 52 | 834 | 88 | 7.3 |
| Pyhäjärvi (Iitti, Kouvola) | SVh | 6105 | 61.02 | 26.48 | 9 | 458 | 28 | 7.3 |
| Pyhäjärvi (Kitee) | SVh | 24764.6 | 61.81 | 29.88 | 6 | 231 | 16 | 7.3 |
| Pyhäjärvi (Lapinjärvi, Orimattila) | Rr | 1310.8 | 60.72 | 26.01 | 58 | 1350 | 110 | 7.8 |
| Pyhäselkä | Sh | 35972.5 | 62.60 | 29.68 | 11 | 395 | 77 | 6.8 |
| Rauanjärvi | MVh | 209.5 | 62.41 | 30.17 | 9 | 285 | 61 | 7 |
| Ruokojärvi | Rk | 154.8 | 64.70 | 29.23 | 21 | 521 | 69 | 7.1 |
| Sääksjärvi | MVh | 155.9 | 62.68 | 26.69 | 13 | 570 | 55 | 7.2 |
| Saaramaanjärvi | Mh | 186.3 | 60.85 | 27.32 | 13 | 390 | 45 | 6.6 |
| Sääskjärvi | Rr | 510.1 | 60.83 | 26.22 | 109 | 921 | 141 | 7.6 |
| Salajärvi | MVh | 404.1 | 61.21 | 26.35 | 5 | 335 | 30 | 6.9 |
| Sälevä | Rh | 1678.2 | 63.52 | 27.88 | 20 | 400 | 100 | 6.2 |
| Saloistenjärvi | Ph | 123.5 | 60.96 | 24.85 | 15 | 570 | 20 | 7.2 |
| Särkijärvi | Vh | 1068.6 | 62.34 | 30.09 | 4 | 162 | 4 | 7.5 |
| Särkkäjärvi | Vh | 62.8 | 62.60 | 31.13 | 2 | 115 | 3 | 7 |
| Savijärvi | Vh | 256.1 | 63.11 | 29.66 | 8 | 510 | 75 | 6.9 |
| Simpelejärvi | SVh | 3322.9 | 61.58 | 29.45 | 11 | 361 | 19 | 7.6 |
| Suolijärvi | Kh | 1095.5 | 65.13 | 28.11 | 21 | 403 | 100 | 6.9 |
| Suomunjärvi | Kh | 670 | 63.14 | 30.75 | 7 | 278 | 80 | 6.4 |
| Suuri-Palonen | Mh | 229.8 | 62.93 | 27.26 | 14 | 420 | 110 | 6.8 |
| Suuri-Pieksä | Vh | 1251.8 | 63.08 | 27.99 | 12 | 464 | 33 | 7.3 |
| Tiilikka | MRh | 425.7 | 63.67 | 28.26 | 10 | 335 | 150 | 5.9 |
| Uurajärvi | Vh | 392.1 | 61.43 | 25.98 | 7 | 300 | 19 | 6.8 |
| Valkeinen | Ph | 147.1 | 63.12 | 27.04 | 9 | 330 | 55 | 6.6 |
| Valvatus | Rk, Rr | 315.5 | 62.22 | 27.83 | 42 | 798 | 122 | 7.4 |
| Viekijärvi | Rh | 2482.1 | 63.41 | 29.72 | 30 | 481 | 150 | 6.5 |
| Viinijärvi | SVh | 3525.9 | 62.65 | 29.40 | 5 | 227 | 7 | 7.2 |
| Viitaanjärvi | Rh | 361.4 | 63.58 | 27.32 | 46 | 664 | 198 | 6.6 |
| Villikkalanjärvi | Rr | 716.8 | 60.77 | 26.04 | 123 | 1451 | 198 | 7.5 |
| Virtasalmen Haapajärvi | MRh | 717.3 | 62.06 | 27.37 | 35 | 605 | 132 | 6 |
| Vuorenselkä | Ph | 91.9 | 61.16 | 24.58 | 65 | 880 | 95 | 7.6 |
